# Editorial: Transcriptional and epigenetic landscapes of abiotic stress response in plants

**DOI:** 10.3389/fpls.2025.1541642

**Published:** 2025-01-20

**Authors:** Aamir W. Khan, Yezhang Ding, Mehanathan Muthamilarasan

**Affiliations:** ^1^ Division of Plant Science and Technology, University of Missouri, Columbia, MO, United States; ^2^ Division of Environmental Genomics and Systems Biology, Lawrence Berkeley National Laboratory, Berkeley, CA, United States; ^3^ Department of Plant Sciences, University of Hyderabad, Hyderabad, India

**Keywords:** transcriptome, epigenetics, abiotic, genomics, DNA methylation

In nature, plants constantly face various biotic and abiotic stresses that impact their growth, development, and productivity. Among these, abiotic stresses often have a more severe impact than biotic stresses. For instance, drought has been reported to cause greater yield losses than the combined impact of all plant pathogens (Gupta et al., 2020). Abiotic stresses are the immediate outcome of climate change, and the magnitude of these stresses has gradually increased every year with the rise in global temperatures. Thus, it has become imperative to study the impact of these stresses on plants and how plants respond to them at different levels to show resilient traits. This includes analysing the plants at morpho-physiological, biochemical, and molecular levels. Researchers often compare stressed plants to control (non-stressed) plants or evaluate contrasting genotypes, such as tolerant and sensitive lines, to elucidate the mechanisms underlying stress responses. While these studies have provided some insights, a comprehensive understanding of the intricate mechanisms governing plant responses to abiotic stress remains largely unknown. Recent advances in next-generation tools and technologies have enabled researchers to dissect the molecular basis of plant stress responses at genomic, transcriptomic, proteomic, metabolomic, epigenetic and epigenomic levels. Among these, knowledge of the transcriptional/epigenomic landscape of the trait-associated variations is limited. Given the importance of transcriptional changes and histone modifications in abiotic stress responses, this Research Topic was edited to collage the knowledge available on transcriptional and epigenetic landscapes of abiotic stress response in plants. The Research Topic features eight original research articles and one review, covering various aspects of transcriptome and epigenetic reprogramming in plants during abiotic stresses. Four of the research articles employ transcriptomics integrated with other omics approaches to explore transcriptome reprogramming, candidate gene identification, and the role of long non-coding RNA during different stresses. Two articles focus on the functional characterization of specific candidate genes involved in stress response, while another provides a genome-wide analysis of a stress-responsive gene family. Additionally, one study investigates genome-wide histone modifications, specifically H3K4me3 and H3K27me3, in response to abiotic stresses.

Wen et al. examined the transcriptional reprogramming in foxtail millet (*Setaria italica*) during foliar application of sodium selenite. The authors showed that selenium (Se) is sequentially transported and accumulated in the leaves, stems, and spikes. Transcriptome analysis revealed significant upregulation of Se metabolism and transporter genes, including those encoding sulfate, phosphate, and nitrate transporters, ABC transporters, antioxidants, phytohormone signaling components, and transcription factors. The study highlighted that these genes interact in complex networks, both synergistically and antagonistically, to regulate selenate transport mechanisms. Further, co-expression network analysis identified the key transcription factors and transporters significantly correlated with Se accumulation and transport. Expression profiling of candidate genes showed the upregulation of genes encoding sulfate transporters (SiSULTR1.2b and SiSULTR3.1a), a phosphate transporter (PHT1.3), a nitrate transporter (NRT1.1B), glutathione S-transferases (GSTs), and an ABC transporter (ABCC13), with an increase in SeO_4_²^-^ accumulation. This study provided comprehensive insights into Se accumulation and transport mechanisms in foxtail millet. While selenium is considered an essential trace element, cadmium (Cd) is recognized as one of the most toxic elements. In a multi-omics study, Wu et al. investigated the physiological, transcriptomic, and metabolomic responses of *Abelmoschus manihot* to Cd stress. Exposure to Cd resulted in significant growth inhibition and oxidative stress in *A. manihot*. Transcriptomic analysis identified differentially expressed genes involved in metal transport, antioxidative defense, and stress signaling pathways. Metabolomic profiling revealed alterations in amino acid metabolism, organic acids, and secondary metabolites, indicating metabolic changes to mitigate Cd toxicity. Integrative analysis highlighted the activation of lipid metabolism and phenylpropanoid biosynthesis pathways, suggesting their roles in Cd detoxification and tolerance. Thus, Wu et al. provided insights into the molecular mechanisms of Cd response in *A. manihot*, which has implications for devising potential strategies for phytoremediation.

The early transcriptional and alternative splicing (AS) responses of tomato (*Solanum lycopersicum*) roots to salt stress were reported by Gan et al. Using DNBSEQ™ technology, Gan et al. identified 3590 differentially expressed genes (DEGs) and 3709 differentially alternatively spliced (DAS) genes during the initial hours of salt exposure. The DEGs were enriched in pathways related to protein metabolism, stress responses, and transcription regulation, indicating a rapid reprogramming of gene expression under salt stress. AS events, particularly exon skipping, were significantly enhanced under salt stress, affecting genes involved in signaling and metabolic processes. The study also reported that genes associated with splicing and spliceosome assembly were differentially expressed, suggesting their role in regulating AS events under stress conditions. These findings expand our knowledge of the molecular mechanisms underlying salt stress response in tomato roots, emphasizing the importance of AS in stress adaptation.

The use of transcriptome sequencing data to study the role of long non-coding RNAs (lncRNAs) was demonstrated by Babaei et al. The authors analyzed the publicly available RNA-seq data of four wheat cultivars (two sensitive and two tolerant cultivars) subjected to heat stress during the pollen development stage. The study identified 11,054 lncRNA-producing loci, with 5,482 lncRNAs showing differential expression in response to elevated temperatures. These heat-responsive lncRNAs potentially regulate protein-coding genes through *cis* and *trans* interactions, as well as within lncRNA-miRNA-mRNA networks. Gene ontology analysis revealed that the target genes of these lncRNAs are involved in processes such as hormonal responses, protein modification and folding, stress responses, and various biosynthetic and metabolic pathways. Specific lncRNA/protein-coding gene pairs and lncRNA-miRNA-mRNA regulatory modules were conserved across multiple cultivars, implicating them in heat stress responses. The study sheds light on lncRNA-mediated regulatory mechanisms during pollen development under heat stress. A similar study on heat stress was conducted by Niu et al., wherein the authors investigated the regulatory role of the SET domain protein SUVH2 in controlling the activity of the heat-activated retrotransposon ONSEN in *A. thaliana*. Under heat stress, ONSEN transcription levels were increased in the *suvh2* mutant; however, no transpositional activity was observed. Notably, the *suvh2* mutant produced small interfering RNAs (siRNAs) from the ONSEN locus during heat stress, indicating that siRNAs suppress transposition. These findings suggest that SUVH2 plays a critical role in the epigenetic regulation of ONSEN through mechanisms involving siRNA-mediated pathways. This study provides evidence of the complex regulatory networks governing retrotransposon activity in plants, particularly under abiotic stress conditions.

C2H2 zinc finger proteins (C2H2-ZFPs) are transcription factors that have critical roles in plant development and stress tolerance. Given this, Du et al. systematically analyzed the C2H2-ZFP gene family in sweet potato (*Ipomoea batatas*), identifying 178 *IbZFP* genes across 15 chromosomes. These proteins were phylogenetically categorized into six clades, and the expansion of this gene family was attributed to 24 tandem and 46 segmental duplications. *In silico* expression profiling of *IbZFP* genes in publicly available RNA-seq data on storage root development highlighted 44 *IbZFP* genes showing differential expression across cultivars. In another dataset on salt stress, 92 *IbZFP* genes showed differential expression to salt stress in salt-tolerant and salt-sensitive varieties. Six *IbZFP* genes were further investigated for tissue-specific and stress-responsive expression under drought, salt, abscisic acid, and gibberellic acid treatments. Further, heterologous expression of *IbZFP105* in *A. thaliana* conferred tolerance to salt stress and increased ABA sensitivity, indicating a positive role of C2H2-ZFPs in stress response. Similarly,Chen et al. examined the impact of overexpressing a ribosome-inactivating protein, OsRIP1, in rice (*Oryza sativa*). OsRIP1 is known to target ribosomal RNA, thereby inhibiting protein synthesis. The authors analyzed the previously developed overexpression lines at phenotypic, transcriptomic, and proteomic levels, in response to methyl jasmonate (MeJA) treatments. Overall, the study suggests that OsRIP1 may antagonize MeJA-induced shoot growth inhibition by modulating cytokinin-mediated leaf senescence and positively regulating cell cycle processes. Interactions between OsRIP1 and proteins, such as the 40S ribosomal protein S5 and α-tubulin, facilitate this modulation. Thus, Chen et al. have provided insights into the complex regulatory roles of OsRIP1 in determining tolerance to exogenous MeJA application in rice.

From an epigenetic perspective, Faivre et al. examined the impact of cold stress on H3K4me3 (associated with gene activation) and H3K27me3 (associated with gene repression) in *A. thaliana*. Under stress conditions, both H3K4me3 and H3K27me3 exhibited rapid and gene-specific redistribution. Interestingly, changes in H3K4me3 and H3K27me3 levels occurred independently on different gene sets, confuting the notion that gene activation under cold stress involves a simple switch from H3K27me3-mediated repression to H3K4me3-mediated activation. Also, the study highlighted a weak correlation between these histone modifications and the changes in the expression of corresponding genes. Altogether, the study provided a genome-wide perspective on cold-triggered histone methylation dynamics, with a lead for further studies on the roles of these marks on corresponding genes.

Finally, a review by Liu et al. summarized the multifaceted roles of auxin response factors (ARFs) in plant development and stress tolerance, emphasizing their functions as transcriptional regulators in auxin signaling. The review provided structural insights into ARF-DNA interactions and explored non-canonical auxin signaling pathways independent of TIR1/AFB-mediated Aux/IAA degradation. This review highlights the versatility of ARFs in regulating organogenesis and abiotic and biotic stress responses.

In conclusion, the Research Topic provided valuable insights into the transcriptional and epigenetic mechanisms underlying abiotic stress responses. Overall, the studies discussed above have provided information on key genes and their potential roles in shared or common pathways and processes regulating stress responses; however, our understanding of the unique and novel mechanisms specific to certain genotypes, species, genera, and families is still limited. Moreover, studies on the effects of multiple or combined stresses on plants are scarce. While it is imperative to encourage more studies on the transcriptional and epigenetic responses of plants to different environmental cues, particularly under multiple or combined stresses, the Research Topic has paved the way for advancing such studies to gain a more comprehensive understanding of plant stress responses.
